# Mitigating Herding in Hierarchical Crowdsourcing Networks

**DOI:** 10.1038/s41598-016-0011-6

**Published:** 2016-12-05

**Authors:** Han Yu, Chunyan Miao, Cyril Leung, Yiqiang Chen, Simon Fauvel, Victor R. Lesser, Qiang Yang

**Affiliations:** 1 Joint NTU-UBC Research Centre of Excellence in Active Living for the Elderly (LILY), Nanyang Technological University,, Singapore, Singapore; 2 School of Computer Science and Engineering, Nanyang Technological University, Singapore, Singapore; 3 Department of Electrical and Computer Engineering, The University of British Columbia, Vancouver, BC Canada; 4 Institute of Computing Technology, Chinese Academy of Sciences, Beijing, China; 5 School of Computer Science, University of Massachusetts Amherst, Amherst, MA USA; 6 Department of Computer Science and Engineering, Hong Kong University of Science and Technology, Hong Kong, China

## Abstract

Hierarchical crowdsourcing networks (HCNs) provide a useful mechanism for social mobilization. However, spontaneous evolution of the complex resource allocation dynamics can lead to undesirable herding behaviours in which a small group of reputable workers are overloaded while leaving other workers idle. Existing herding control mechanisms designed for typical crowdsourcing systems are not effective in HCNs. In order to bridge this gap, we investigate the herding dynamics in HCNs and propose a Lyapunov optimization based decision support approach - the Reputation-aware Task Sub-delegation approach with dynamic worker effort Pricing (RTS-P) - with objective functions aiming to achieve superlinear time-averaged collective productivity in an HCN. By considering the workers’ current reputation, workload, eagerness to work, and trust relationships, RTS-P provides a systematic approach to mitigate herding by helping workers make joint decisions on task sub-delegation, task acceptance, and effort pricing in a distributed manner. It is an individual-level decision support approach which results in the emergence of productive and robust collective patterns in HCNs. High resolution simulations demonstrate that RTS-P mitigates herding more effectively than state-of-the-art approaches.

## Introduction

The organization of social and economic activities to efficiently coordinate participants’ effort is an important topic of economic theory. Thanks to the Internet, social media and online social networks, social mobilization through crowdsourcing has achieved unprecedented success. Crowdsourcing refers to the process whereby clients (a.k.a. *crowdsourcers*) obtain needed services by soliciting contributions from a large group of people (a.k.a. *workers*)^1^. Crowdsourcing communities based around social networks tend to have hierarchical structures^2,3^. These hierarchical crowdsourcing networks (HCNs) have been used to mobilize the masses in many significant real-world applications including political rallies^4^, scientific research^5^, mapping out natural environment features^6,7^, and large-scale search-and-rescue missions^8^.

In essence, crowdsourcing systems can be treated as resource allocation ecosystems containing a large number of interacting workers (i.e., resources) and crowdsourcers. Crowdsourcers are typically self-interested; their primary intention is to maximize their own utilities. This will usually lead them to only select workers with high perceived reputation, leading to the emergence of *herding* ^9^. Herding refers to the situation in which a large number of task requests concentrate on a small group of reputable workers, causing them to be overloaded while leaving other workers idle. It can lead to cascading failures and eventually result in catastrophic system breakdown^10^. The risk of herding is especially pronounced in HCNs in which crowdsourcers lack global knowledge and workers have limited resources to be tapped into^11^.

Mitigating herding in HCNs is important to ensure sustainable operation of these problem solving ecosystems^12^. In general, workers in an HCN make three important decisions in a distributed manner: 1) *how much new workload to accept*, 2) *how much existing workload to sub-delegate to others in the HCN (and to whom)*, and 3) *how to price their services*. The collective effect of these joint decisions made by all HCN participants determines whether herding will emerge. Therefore, herding mitigation mechanisms need to influence these three decisions by each worker in order to improve the overall efficiency of an HCN. The human nature of the HCN participants imposes additional complexities on this already challenging problem:

1. **Worker heterogeneity**: Workers have different skill levels and productivity. They may produce results of different quality when assigned the same task, and may not be able to maintain the same level of productivity everyday.
2. **Timing and targets for sub-delegation**: It is difficult for a worker to quantify when sub-delegation is needed and who the suitable candidates for sub-delegation are. This is further complicated by the fact that different workers may incur different costs to complete the same task. Sub-delegation to a worker resulting in a loss for the sub-delegator is not a rational choice.
3. **Workers’ commitment**: Workers may not be fully committed to an HCN. Their eagerness to work (which may change over time) will affect their availability.

**Figure 1:**
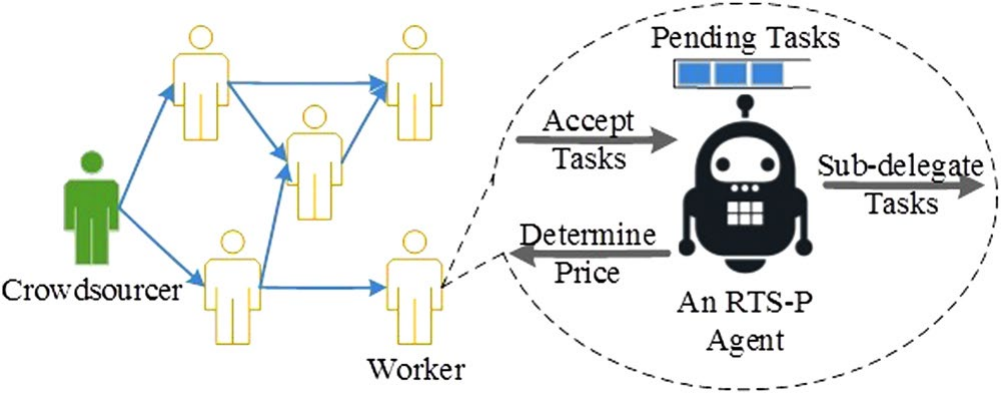
An RTS-P agent in an HCN.

Recently, computational approaches for mitigating herding in crowdsourcing systems have emerged. In the Pinning control method^10^, the pinning method is used to control the collective dynamics in complex networks. The study focuses on situations where multiple agents try to decide individually which one of two available resources to use. Thus, this method cannot be directly applied to crowdsourcing systems in which many crowdsourcers need to engage a large number of workers to accomplish their objectives. The Global Considerations (GC) approach uses a worker’s current pending workload as a guide to adjust his reputation^13^. GC adjusts the probability for a task to be assigned to a worker based on the worker’s reputation standing among all other workers using the *softmax* approach. In Yu *et al.* ^14^, a centralized task allocation approach was proposed to make dynamic trade-offs between the need for engaging trustworthy workers and obtaining task results on time. A fully distributed variant of this method that helps workers determine which incoming tasks to accept was studied in Yu *et al.* ^15^. All of these approaches allow workers to be automatically assigned to tasks, saving them time spent on exploring open task calls and improving their collective productivity. Nevertheless, these existing approaches are not designed for HCNs. They do not support task sub-delegation, an essential mechanism to avoid herding in HCNs. The aforementioned complexities due to human nature have also not been accounted for by existing approaches.This paper investigates the herding dynamics in HCNs and proposes the Reputation-aware Task Sub-delegation approach with dynamic worker effort Pricing (RTS-P) to mitigate herding through enhancing the efficiency of manpower utilization in HCNs. It is an individual-level decision-making approach based on Lyapunov optimization^16^ with objective functions aiming to achieve superlinear time-averaged collective productivity in an HCN^17^. By considering a worker’s current reputation, workload, willingness to work, and his trust relationships with others, RTS-P provides a systematic approach for a worker to make joint decisions on task acceptance, sub-delegation, and effort pricing, so as to maximize his income while avoiding significant fluctuations in workload. The approach is distributed and can be implemented as a personal decision support agent for a worker in an HCN (Fig. 1). RTS-P is an extension of our previous model - RTS^18^. The addition of the dynamic worker effort pricing function allows operation in systems which permit workers to set the price of their service. In doing so, substantial modifications to the original system model^18^ and the joint task acceptance and sub-delegation decisions are required.

RTS-P is compared with 4 existing methods through extensive experiments based on a large-scale real-world dataset - the *Epinions* trust network dataset. The results show that RTS-P effectively mitigates herding through efficiently harnessing the available human resources. We also show that RTS-P workers achieve significantly higher total income compared with other state-of-the-art approaches, especially under high workload conditions. RTS-P not only automates key decisions in the situation-task-others triad^19^ surrounding a worker, but also sheds light on the long-standing quest for an individual-level decision support approach which results in productive and robust collective patterns in human crowds^20^. Our work provides a general framework to optimally harness the collective productivity of a complex network of human resources in order to mitigate herding, with potential applications in many social and economic systems.

## Methods

Our key results include (1) a formulation of the problem of mitigating herding through efficiently harnessing the productivity of workers in an HCN as a constrained optimization problem which minimizes drastic fluctuations in workers’ workloads while maximizing their expected earnings; (2) a distributed algorithm which solves the problem by jointly controlling the task acceptance, task sub-delegation, and effort pricing decisions for each worker; and (3) experimental evaluations of the performance of the proposed algorithm against state-of-the-art approaches in a large-scale HCN.

### Proposed Framework

Our focus in this paper is to address the problem of delegating/sub-delegating a task, *τ*_*j*_, proposed by a crowdsourcer *j*, to workers in an HCN. In general, the effort required to complete a task (i.e. the workload of the task) can be expressed in *effort units* which can be defined by crowdsourcing system operators. For example, the effort required to complete a software programming task can be measured by the expected number of lines of code. A task must be completed before its stipulated deadline and with quality acceptable to the crowdsourcer. A worker *i* has a limited effort output rate which can be up to ${\mu }_{i}^{{\rm{\max }}}$ effort units per time slot. Tasks waiting to be completed by *i* are stored in his pending tasks queue. Let *q*_*i*_(*t*) be worker *i*’s pending workload at the beginning of time slot *t*; the queuing dynamics of *q*_*i*_(*t*) can be formulated as:

1 $${q}_{i}(t+{\rm{1}})=max\,[{q}_{i}(t)+{\lambda }_{i}(t)\,-{\mu }_{i}(t)\,-{s}_{i}(t),\,{\rm{0}}]$$

where *λ*_*i*_(*t*) is the new workload accepted into *q*_*i*_(*t*) during time slot *t*, *μ*_*i*_(*t*) represents the actual workload completed by *i* during time slot *t*, and *s*_*i*_(*t*) is the sub-delegated workload by worker *i* during time slot *t*.

With crowdsourcing analytics tools such as Turkalytics^21^, workers’ performance can be tracked in detail. A worker *i*’s past performance as measured by the quality and timeliness of his productive output can be used to estimate *i*’s reputation, *r*_*i*_(*t*) ∈ (0, 1), using a reputation evaluation model^22^. Reputation acts as a sanctioning mechanism affecting future demand for a worker’s services. Using this information, a worker can establish trust relationships with a set of other known workers, ${{\bf{n}}}_{i}^{sub}$. ${{\bf{n}}}_{i}^{sub}$ is the set of trusted workers to whom worker *i*’s tasks can potentially be delegated or sub-delegated. As a task can be iteratively sub-delegated through a *delegation chain*, it is reasonble for all workers in the delegation chain to be accountable (to various degrees) for the outcome of the task. A possible model for sharing responsibility in a delegation chain is the *Decreasing Weighting* (DW) reputation update mechanism^23^ which assigns the last worker in the delegation chain (i.e. the one who actually completed the task) the highest share of responsibility and decreasing weight values to other workers higher up along the delegation chain. The future expected demand for a worker *i*’s service, ${\mathbb{E}}\{{\lambda }_{i}(t)\}$, is affected by his *r*_*i*_(*t*) value and the current price he charges for his service, *p*_*i*_(*t*) (e.g., measured in dollars per effort unit), i.e.,

2 $${\mathbb{E}}\{{\lambda }_{i}(t)\}=f({p}_{i}(t),{r}_{i}(t)).$$

#### Automating Task Sub-delegation Decisions

An RTS-P agent takes only local knowledge as input, and automatically offers recommendations to a worker *i* concerning three key decisions in an HCN at any given point in time: 1) the timing, amount of workload, and the target workers for sub-delegation, 2) how much new workload shall be accepted by *i*, and 3) how to price his services. If an RTS-P agent determines that its owner *i*’s risk of not completing all pending tasks before the respective deadlines is high, it will attempt to sub-delegate some of the pending tasks to other workers. The selection of candidate workers for sub-delegation takes into account how trusted the workers are and how much they charge for their services (i.e., so that the act of sub-delegating does not incur financial loss for its owner). These heuristics can be converted into a computational task sub-delegation mechanism as follows.

A conceptual queue, *Q*_*i*_(*t*), is used to quantify the urgency for a worker *i* to sub-delegate pending tasks. *Q*_*i*_(*t*) is updated by an RTS-P agent in conjunction with *q*_*i*_(*t*) as follows:

(3) $${Q}_{i}(t+\mathrm{1})=\,{\rm{\max }}[{Q}_{i}(t)-{\mu }_{i}(t)-{s}_{i}(t)+{\bar{\lambda }}_{i}{1}_{[{q}_{i}(t)> \mathrm{0}]},\mathrm{0}].$$

In this formulation, the symbol ${\bar{\lambda }}_{i}$ represents the average amount of new workload accepted by worker *i* per time slot. 1_[condition]_ is an indicator function. Its value is 1 if and only if [condition] is satisfied; otherwise, it evaluates to 0. The dynamics of *Q*_*i*_(*t*) are as follows:

- The workload in *Q*_*i*_(*t*) is increased in such way that if *q*_*i*_(*t*) is non-empty at the time when the value of *Q*_*i*_(*t*) is updated, then *Q*_*i*_(*t*) grows by ${\bar{\lambda }}_{i}$. This ensures that *Q*_*i*_(*t*) keeps increasing if there are tasks in *q*_*i*_(*t*) which have not been completed for some time.
- The value of *Q*_*i*_(*t*) is reduced by the task servicing process [−*μ*_*i*_(*t*) − *s*_*i*_(*t*)].

In order to efficiently utilize the productivity of a crowdsourcing network, RTS-P must ensure that the upper bounds of both *q*_*i*_(*t*) and *Q*_*i*_(*t*) are finite for all workers involved.

Let *X*_*i*_(*t*) = (*q*_*i*_(*t*), *Q*_*i*_(*t*)) be a concatenated vector of worker *i*’s physical and conceptual pending tasks queues. We adopt the *Lyapunov* function^16^ to measure the level of congestion in both *q*_*i*_(*t*) and *Q*_*i*_(*t*) for all workers in a given HCN. It can be expressed as $L({X}_{i}(t))=\frac{1}{2}[{q}_{i}^{2}(t)+{Q}_{i}^{2}(t)]$. Then, the amount of change in worker *i*’s pending workload can be measured using the conditional *Lyapunov* drift as:

4 $${\rm{\Delta }}({X}_{i}(t))={\mathbb{E}}\{L({X}_{i}(t+1))\,-\,L({X}_{i}(t))| {X}_{i}(t)\}={\mathbb{E}}\{[\frac{1}{2}{q}_{i}^{2}(t+1)+\frac{1}{2}{Q}_{i}^{2}(t+1)]\,-\,[\frac{1}{2}{q}_{i}^{2}(t)+\frac{1}{2}{Q}_{i}^{2}(t)]| {X}_{i}(t)\}={\mathbb{E}}\{\frac{1}{2}{q}_{i}^{2}(t+1)\,-\,\frac{1}{2}{q}_{i}^{2}(t)+\frac{1}{2}{Q}_{i}^{2}(t+1)\,-\,\frac{1}{2}{Q}_{i}^{2}(t)| {X}_{i}(t)\}.$$

Based on equation (3), we have:

5 $$\begin{array}{rcl}\frac{1}{2}{Q}_{i}^{2}(t+1)-\frac{1}{2}{Q}_{i}^{2}(t) & = & \frac{1}{2}{[{\bar{\lambda }}_{i}-({\mu }_{i}(t)+{s}_{i}(t))]}^{2}+{Q}_{i}(t)[{\bar{\lambda }}_{i}-{\mu }_{i}(t)-{s}_{i}(t)]\\  & = & \frac{1}{2}\{{({\bar{\lambda }}_{i})}^{2}-2{\bar{\lambda }}_{i}[{\mu }_{i}(t)+{s}_{i}(t)]+{[{\mu }_{i}(t)+{s}_{i}(t)]}^{2}\}\\  &  & +{Q}_{i}(t)[{\bar{\lambda }}_{i}-{\mu }_{i}(t)-{s}_{i}(t)]\\  & \leq  & \frac{1}{2}[{({\lambda }_{i}^{{\rm{\max }}})}^{2}+{({\mu }_{i}^{{\rm{\max }}}+{s}_{i}^{{\rm{\max }}})}^{2}]+{Q}_{i}(t)[{\bar{\lambda }}_{i}-{\mu }_{i}(t)-{s}_{i}(t)]\end{array}$$

where ${\lambda }_{i}^{{\rm{\max }}}$ and ${s}_{i}^{{\rm{\max }}}$ are the respective upper bounds of *λ*_*i*_(*t*) and *s*_*i*_(*t*) for a given worker *i*.

Based on the same approach, the conditional Lyapunov drift for the physical queue can be expressed as:

6 $$\frac{1}{2}{q}_{i}^{2}(t+1)-\frac{1}{2}{q}_{i}^{2}(t)\,\leq \,\frac{1}{2}[{({\lambda }_{i}^{{\rm{\max }}})}^{2}+{({\mu }_{i}^{{\rm{\max }}}+{s}_{i}^{{\rm{\max }}})}^{2}]+{q}_{i}(t)[{\lambda }_{i}(t)-{\mu }_{i}(t)-{s}_{i}(t)].$$

From equations (5) and (6), equation (4) can be expressed as:

7 $${\rm{\Delta }}({X}_{i}(t))\leq {\mathbb{E}}\{{({\lambda }_{i}^{max})}^{2}+{({\mu }_{i}^{max}+{s}_{i}^{max})}^{2}+{Q}_{i}(t)[{\mathop{\lambda }\limits^{\bar{\i}}}_{i}-{\mu }_{i}(t)\,-{s}_{i}(t)]+{q}_{i}(t)[{\lambda }_{i}(t)\,-{\mu }_{i}(t)\,-{s}_{i}(t)]| {X}_{i}(t)\}.$$

For simplicity of notation, let ${C}_{i}={({\lambda }_{i}^{{\rm{\max }}})}^{2}+{({\mu }_{i}^{{\rm{\max }}}+{s}_{i}^{{\rm{\max }}})}^{2}$. From a worker *i*’s view point, he would wish to minimize both the cost incurred by task sub-delegation as well as drastic changes in his pending workload. Thus, we formulate a {*drift* + *cost*} expression to capture this dual goal as follows:

8 $${\rm{\Delta }}({X}_{i}(t))+{\rho }_{i}(t){\mathbb{E}}\{{\varphi }_{i}(t){s}_{i}(t)| {X}_{i}(t)\}\phantom{\rule{0ex}{0ex}}\leq {C}_{i}+{Q}_{i}(t){\mathbb{E}}\{{\mathop{\lambda }\limits^{\bar{\i}}}_{i}-{\mu }_{i}(t)\,-{s}_{i}(t)| {X}_{i}(t)\}+{q}_{i}(t){\mathbb{E}}\{{\lambda }_{i}(t)-{\mu }_{i}(t)\,-{s}_{i}(t)| {X}_{i}(t)\}+{\rho }_{i}(t){\mathbb{E}}\{{\varphi }_{i}(t){s}_{i}(t)| {X}_{i}(t)\}.$$

where ${\varphi }_{i}(t)=\frac{1}{|{{\bf{n}}}_{i}^{sub}|}{\sum }_{k\in {{\bf{n}}}_{i}^{sub}}{p}_{k}(t)$ represents the average price of service charged by worker *i*’s known trusted workers at time slot *t*, and *ρ*_*i*_(*t*) > 0 represents *i*’s general eagerness to work. A large value of *ρ*_*i*_(*t*) indicates that a worker is highly motivated to work. It adjusts the relative importance given to the two components in the {*drift* + *cost*} expression. It can be inferred by keeping track of the worker’s productivity over a period of time, or be explicitly declared by the worker to control how the RTS-P agent behaves.

At the beginning of each time slot, the RTS-P agent observes *q*_*i*_(*t*) and *Q*_*i*_(*t*), as well as its owner *i*’s current context tuple 〈*μ*_*i*_(*t*), *λ*_*i*_(*t*), *φ*_*i*_(*t*)〉, to determine the value of *s*_*i*_(*t*) which minimizes the {*drift* + *cost*} expression. This form of combined value maximization and surprise minimization complies with the latest findings in human choice behaviours^24^. By only considering the terms containing the decision variable *s*_*i*_(*t*) which can be controlled by the RTS-P agent in equation (8), the {*drift* + *cost*} objective function can be re-expressed as:

Minimize:

9 $$\frac{1}{T}\sum _{t=0}^{T-1}\sum _{i=1}^{N}{s}_{i}(t)[{\rho }_{i}(t){\varphi }_{i}(t)-{q}_{i}(t)-{Q}_{i}(t)]$$

Subject to:

10 $$0\,\leq \,{s}_{i}(t)\,\leq \,{q}_{i}(t)$$

11 $$\exists k\in {{\bf{n}}}_{i}^{sub},\exists {\tau }_{j}\in {q}_{i}(t),{r}_{k}(t)\geq {r}_{{\rm{\min }}}(t)\wedge {p}_{k}(t)\,\leq \,{p}_{{\tau }_{j}}.$$

${p}_{{\tau }_{j}}$ is the price the worker *i* charges for task *τ*_*j*_. *r*_min_(*t*) ∈ [0, 1] is a pre-determined reputation threshold value. In order to minimize equation (9), ${\hat{s}}_{i}(t)$, the target value of *s*_*i*_(*t*), is:

12 $${\hat{s}}_{i}(t)=\left\{\begin{array}{ll} 0, \quad {\rm{if}}\,{\rho }_{i}(t){\varphi }_{i}(t)-{q}_{i}(t)-{Q}_{i}(t) \geq 0\\ {q}_{i}(t)-{\mu }_{i}(t),\quad {\rm{otherwise}}.\end{array}\right.$$

Intuitively, equation (12) means that when worker *i* is highly willing to work, the cost of sub-delegating is high, the current workload is low, and tasks in the pending tasks queue have not been pending for too long, worker *i* should not sub-delegate any tasks. Otherwise, worker *i* should try to sub-delegate as many tasks as possible. Nevertheless, the actual *s*_*i*_(*t*) value also depends on the satisfaction of Constraint (11), which requires at least one worker *k* in ${{\bf{n}}}_{i}^{sub}$ whose reputation is higher than the threshold, and who charges a price no higher than what worker *i* charges for the task (i.e., worker *i* does not incur any loss by sub-delegating the task to worker *k*).

#### Automating Task Acceptance and Effort Pricing Decisions

Taking the cost of task sub-delegation into account, the expected income for a worker *i* at time slot *t* becomes ${\mathbb{E}}\{{\xi }_{i}(t)| {p}_{i}(t),{r}_{i}(t)\}={a}_{i}(t){p}_{i}(t){r}_{i}(t)f({p}_{i}(t),{r}_{i}(t))\,-{\varphi }_{i}(t){s}_{i}(t)$ where *a*_*i*_(*t*) is a binary decision variable which controls if worker *i* accepts new tasks at the beginning of time slot *t*. In this case, a worker *i* would wish to maximize his income while minimizing drastic fluctuations in his pending workload. Similar to equation (8), this objective function can be formulated as an {*income − drift*} function:

13 $$\begin{array}{rcl}{\rho }_{i}(t){\mathbb{E}}\left\{{\xi }_{i}(t)|{X}_{i}(t)\right\}-{\rm{\Delta }}({X}_{i}(t)) & \leq  & {\rho }_{i}(t){\mathbb{E}}\left\{{a}_{i}(t){p}_{i}(t){r}_{i}(t)f({p}_{i}(t),{r}_{i}(t))\right.\\  &  & \left.-{\varphi }_{i}(t){s}_{i}(t)|{X}_{i}(t)\right\}-{C}_{i}-{q}_{i}(t){\mathbb{E}}\left\{{a}_{i}(t)\right.\\  &  & \left.\times \,f({p}_{i}(t),{r}_{i}(t))-{\mu }_{i}(t)-{s}_{i}(t)|{X}_{i}(t)\right\}\\  &  & -{Q}_{i}(t){\mathbb{E}}\{{\bar{\lambda }}_{i}-{\mu }_{i}(t)-{s}_{i}(t)|{X}_{i}(t)\}\end{array}$$

which is to be maximized.

A recent large scale empirical study in e-commerce, involving sellers from both eBay and Taobao^25^, suggests the following expression relating new demand (i.e., workload) for a worker to his price and reputation:

14 $$\mathrm{ln}\,f({p}_{i}(t),{r}_{i}(t))={c}_{0}-{c}_{1}\,\mathrm{ln}\,{r}_{i}(t)+{c}_{2}\,\mathrm{ln}\,{N}_{i}^{p}(t)+{c}_{3}{d}_{i}+\,\mathrm{ln}\,{p}_{i}(t)$$

where *c*_0_ to *c*_3_ are positive constants, ${N}_{i}^{p}(t)$ is the number of positive ratings received by *i* over a given period of time, and *d*_*i*_ represents how similar the quality of service provided by *i* is to what he promises. In this paper, we adopt equation (14) for modeling the dynamics of the demand for a worker’s service to derive the joint task acceptance and effort pricing strategy. Nevertheless, equation (14) can be replaced by other functions suitable for different systems without affecting the principle on which RTS-P operates.

By taking exponents on both sides of equation (14), we have:

15 $$f({p}_{i}(t),{r}_{i}(t))={\vartheta }_{i}\frac{{p}_{i}(t)}{{[{r}_{i}(t)]}^{{c}_{1}}}$$

where ${\vartheta }_{i}={e}^{({c}_{0}+{c}_{3}{d}_{i})}{[{N}_{i}^{p}(t)]}^{{c}_{2}}$. As RTS-P only controls the decision variables *p*_*i*_(*t*) and *a*_*i*_(*t*) for effort pricing and task acceptance, we only consider the terms containing these decision variables on the right hand side of equation (13) and substitute *f*(*p*_*i*_(*t*), *r*_*i*_(*t*)) with equation (15). Thus, we have:

Maximize:

16 $$\frac{1}{T}\sum _{t=0}^{T-1}\sum _{i=1}^{N}\frac{{a}_{i}(t){\vartheta }_{i}{p}_{i}(t)}{{[{r}_{i}(t)]}^{{c}_{1}}}[{p}_{i}(t){r}_{i}(t){\rho }_{i}(t)-{q}_{i}(t)]$$

Subject to:

17 $${p}_{i}(t)\,\geq \,{p}_{i}^{min},\forall t,\forall i$$

where ${p}_{i}^{{\rm{\min }}}$ is the minimum price to cover *i*’s cost of service. We assume the value of ${p}_{i}^{{\rm{\min }}}$ does not change frequently and can be treated as a constant with respect to *i*. The solution for maximizing this objective function can be obtained by finding the first order derivative of equation (16) and equating it to 0:

18 $$\frac{d}{d{p}_{i}(t)}\{\frac{{a}_{i}(t){\vartheta }_{i}}{{[{r}_{i}(t)]}^{{c}_{1}}}[{r}_{i}(t){\rho }_{i}(t){p}_{i}^{2}(t)-{q}_{i}(t){p}_{i}(t)]\}=0.$$

Solving equation (18) yields:

19 $${p}_{i}(t)=\,{\rm{\max }}[{p}_{i}^{{\rm{\min }}},\frac{{q}_{i}(t)}{2{\rho }_{i}(t){r}_{i}(t)}]\mathrm{.}$$

The result means that *i* should increase the price he charges for new tasks if his current workload is high, his current reputation is low, or his eagerness to work is low (and vice versa), while ensuring that his price is always no less than ${p}_{i}^{{\rm{\min }}}$. If *i*’s reputation is low, he is less likely to receive a large number of task requests. Thus, whenever others are willing to solicit *i*’s service, from *i*’s perspective, he should charge a higher price in order to capitalize on these opportunities.

To maximize equation (16):

20 $${a}_{i}(t)=\left\{\begin{array}{ll}1, & {\rm{if}}\,{p}_{i}(t){r}_{i}(t){\rho }_{i}(t)-{q}_{i}(t)> 0\\ \mathrm{0}, & {\rm{otherwise}}.\end{array}\right.$$

In this paper, we ensure that a worker is never assigned more workload than the maximum workload he can handle within one time slot. Thus, when *a*_*i*_(*t*) = 1, RTS-P accepts up to ${\mu }_{i}^{{\rm{\max }}}$ effort units worth of new workload into *i*’s pending tasks queue.

The core RTS-P algorithm is presented in Algorithm 1. It can be implemented as a personal decision support agent for each worker in an HCN. Multiple RTS-P agents can then communicate on their respective owners’ behalf to automate the task acceptance, sub-delegation, and pricing decisions to maximize the overall productivity of the given crowdsourcing network.

**Table:** 

**Algorithm 1** RTS-P
**Require:** *q*_*i*_(*t*), *Q*_*i*_(*t*), ${p}_{i}^{min}$, *r*_*i*_(*t*), *r*_min_(*t*), *ρ*_*i*_(*t*), *φ*_*i*_(*t*), and the amount of new tasks demanding worker *i*’s service at time slot *t*, ${q}_{i}^{new}(t)$.
1: $${p}_{i}(t)\leftarrow \,max\,[{p}_{i}^{min},\frac{{q}_{i}(t)}{2{\rho }_{i}(t){r}_{i}(t)}]$$;
2: **if** *a*_*i*_(*t*) = 1 **then**
3: **if** $${q}_{i}^{new}(t) > {\mu }_{i}^{max}$$ **then**
4: $${\lambda }_{i}(t)\leftarrow {\mu }_{i}^{max}$$
5: **else**
6: $${\lambda }_{i}(t)\leftarrow {q}_{i}^{new}(t)$$ (t);
7: **end if**
8: **else**
9: *λ*_*i*_(*t*) ← 0;
10: **end if**
11: Return the $[{q}_{i}^{new}(t)\,-{\lambda }_{i}(t)]$ unaccepted tasks to the original sub-delegator(s);
12: **if** $${\rho }_{i}(t){\varphi }_{i}(t)\,-{q}_{i}(t)\,-{Q}_{i}(t)\geq 0$$ **then**
13: *s*_*i*_(*t*) ← 0;
14: **else**
15: *s*_*i*_(*t*) ← *q*_*i*_(*t*) − *μ*_*i*_(*t*);
16: **for** each worker *k* in ${n}_{i}^{sub}$ with ${r}_{k}(t)\geq {r}_{min}(t)$ **do**
17: Set *d*_*k*_(*t*) to denote the amount of workload from sub-delegating tasks in *s*_*i*_(*t*) which satisfies ${p}_{k}(t)\leq {p}_{{\tau }_{j}}$;
18: **if** *d*_*k*_(*t*) > 0 **then**
19: *u*_*k*_(*t*) ← Invoking worker *k*’s RTS-P agent with ${q}_{k}^{new}(t)+={d}_{k}(t)$;
20: **end if**
21: *s*_*i*_(*t*)− = [*d*_*k*_(*t*) − *u*_*k*_(*t*)];
22: **if** *s*_*i*_(*t*) = 0 **then**
23: Exit the **for** loop;
24: **end if**
25: **end for**
26: **end if**
27: Update *q*_*i*_(*t* + 1) following equation (1)
28: Update *Q*_*i*_(*t* + 1) following equation (3)

In our previous work^18^, we have proved that the joint task acceptance and sub-delegation decisions made under prices of service fixed by the crowdsourcers are asymptotically optimal compared to an oracle that knows the exact situation of each worker at all times. Although the addition of dynamic worker effort pricing in RTS-P allows workers to adjust their prices according to their changing situations, the joint task acceptance and sub-delegation decisions are made only after the prices have been set. Thus, the original theoretical analysis is still valid for RTS-P. Interested readers may refer to the Analysis section in Yu *et al.* ^18^.

## Results

To evaluate the performance of RTS-P under realistic settings, it is compared with four state-of-the-art approaches through extensive numerical experiments in an HCN based on the *Epinions* trust network dataset^26^. This real-world dataset allows us to construct realistic scenarios for performance comparison. The simulations facilitate understanding of the behavior of RTS-P under different situations.

### Model Implementation on a Real Network

The *Epinions* trust network dataset used in the experiments contains *N* = 10,476 workers, each represented by a node in the network structure. These nodes are connected by weighted and directed edges. A weight of “+1” represents a trust relationship, while a weight of “−1” represents a distrust relationship. The dataset contains 15,742 trust relationships and 2,170 distrust relationships. Based on this dataset, we construct an HCN populated by worker agents with different characteristics. For a worker agent *i* in the experiment, ${{\bf{n}}}_{i}^{sub}$ consists of other worker agents connected with *i* through a directed “+1” edge originating from *i*. We assume that agents do not have global awareness. Thus, a worker agent *i* may only delegate or sub-delegate tasks to other worker agents in ${{\bf{n}}}_{i}^{sub}$. Each worker agent *i* has an innate trustworthiness *h*_*i*_ ∈ [0, 1] which dictates its probability of producing satisfactory results for tasks delegated to it in simulations. This value is computed using the number of other agents trusting and distrusting agent *i* in the dataset following the Beta Reputation Model^27^.

**Figure 2:**
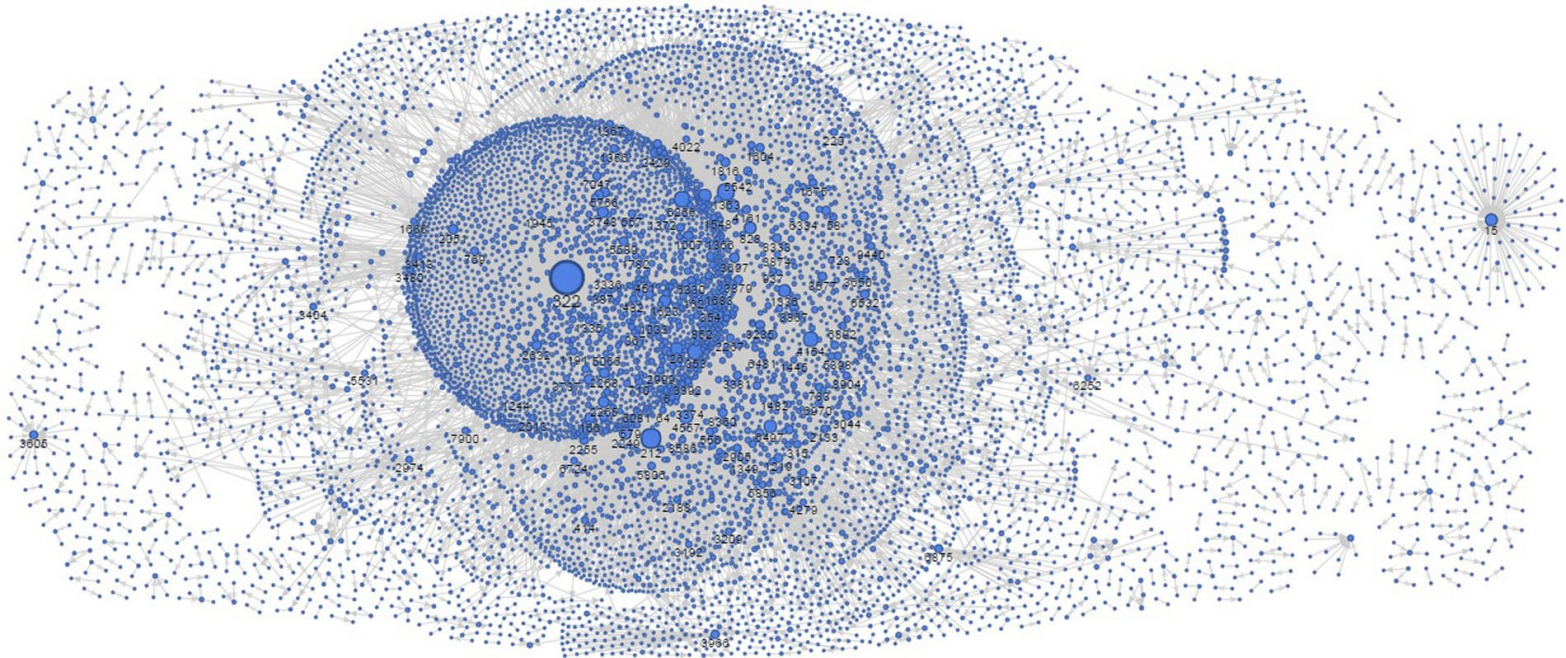
The HCN based on the *Epinions* trust network structure. Nodes represent worker agents and arrows represent trust relationships. The larger the size of a node, the more trusted a worker agent is.

Figure 2 illustrates the crowdsourcing network derived from the dataset. The size of a node in the figure reflects the worker agent’s *h*_*i*_ value. The larger the size of a node, the more trusted the worker agent is. Let *ρ* be the workers’ average eagerness to work in a given crowdsourcing network:

21 $$\rho =\frac{1}{N}\sum _{i=1}^{N}{\rho }_{i}(t).$$

The value of *ρ* is varied from 1 to 100 to simulate different levels of workers’ general eagerness to work.

The relationship between worker agents’ ${\mu }_{i}^{max}$ values and *h*_*i*_ values is varied in three different ways as denoted by a setting variable ${R}_{\mu }^{h}\in \{-,\,{\rm{0,}}\,+\}$. Under ${R}_{\mu }^{h}=-$, ${\mu }_{i}^{{\rm{\max }}}$ values are inversely proportional to *h*_*i*_ values (i.e., workers who produce high quality results have small task processing capacities). Under ${R}_{\mu }^{h}=0$, ${\mu }_{i}^{{\rm{\max }}}$ values are independent from *h*_*i*_ values (i.e., workers’ task processing capacities are not related to the quality of their work). Under ${R}_{\mu }^{h}=+$, ${\mu }_{i}^{{\rm{\max }}}$ values are directly proportional to *h*_*i*_ values (i.e., workers who produce high quality results have large task processing capacities). These settings are created to simulate different worker behaviour characteristics to study how effectively RTS-P copes with these situations. Files containing the HCNs used in the experiments can be downloaded from http://goo.gl/QyRjTs.

In the experiments, we assume that the outcome for each task is binary (i.e., a task is regarded to be *successful* if the worker agent produces the correct result before the stipulated deadline; otherwise, it is considered *unsuccessful*). A worker agent is only paid if it completes a task successfully. Five duplicate crowdsourcing networks are created in the experiments to study the relative performance of the 5 approaches. They are:

1. The Equality-based Approach (EA): tasks are uniformly distributed among worker agents in a crowdsourcer agent *j*’s ${{\bf{n}}}_{j}^{sub}$ without regard to their reputations.
2. The Reputation-based Approach (RA): the probability for a worker agent *i* to be selected by a crowdsourcer agent *j* is determined by its reputation standing among all worker agents in ${{\bf{n}}}_{j}^{sub}$ following the softmax choice rule^28^.
3. The Global Considerations (GC) Approach: a crowdsourcer agent *j* adjusts the probability for tasks to be delegated to each worker agent in ${{\bf{n}}}_{j}^{sub}$ following the approach in Grubshtein *et al*.^13^.
4. The DRAFT Approach: worker agents make task request acceptance decisions following the approach in Yu *et al.* ^15^.
5. The RTS-P Approach: worker agents follow the approach proposed in this paper.

Approaches 1 to 4 do not support task sub-delegation.

The overall workload level in the experimental HCN is adjusted to simulate different operational conditions. As the workload is measured in relative terms to the collective task processing capacity of the worker agents, we compute the maximum throughput *θ* of a given crowdsourcing network as $\theta ={\sum }_{i=1}^{N}{h}_{i}{\mu }_{i}^{{\rm{\max }}}$. At each time step, a proportion of the agents, *p*_*a*_, from the network are selected at random to act as crowdsourcers from which tasks originate. Based on empirical studies of the mTurk crowdsourcing system^29,30^, the ratio between crowdsourcers and workers is close to 1:20. Thus, we set *p*_*a*_ = 5%. The workload for a given crowdsourcing network is measured by the *Load Factor* (LF). It is calculated as $LF=\frac{{w}_{req}}{\theta }$, where *w*_*req*_ is the amount of new workload generated by crowdsourcer agents at each time slot. In the experiments, the *LF* value ranges from 5% to 100% in 5% increments. Under each *LF* setting, the simulation is run for *T* = 10,000 time slots. Task deadlines are randomized. On average, a task must be completed within 5 time slots after it is first assigned to a worker agent.

### Simulation Results

**Figure 3:**
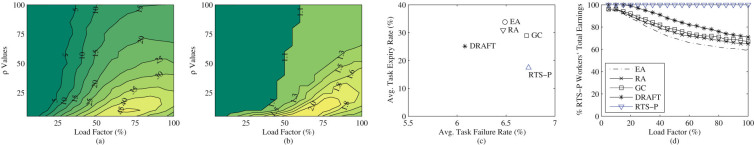
Experimental results under the worker behaviour characteristic setting ${R}_{\mu }^{h}=+$: (**a**) The percentage of all tasks successfully sub-delegated by RTS-P agents; (**b**) The average sub-delegation chain length; (**c**) The average task expiry rates vs. the average task failure rates; (**d**) The total income as a percentage of the total income of RTS-P agents.

**Figure 4:**
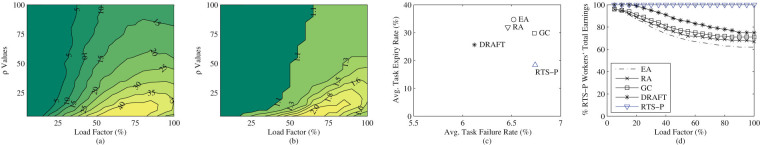
Experimental results under the worker behaviour characteristic setting ${R}_{\mu }^{h}=0$: (**a**) The percentage of all tasks successfully sub-delegated by RTS-P agents; (**b**) The average sub-delegation chain length; (**c**) The average task expiry rates vs. the average task failure rates; (**d**) The total income as a percentage of the total income of RTS-P agents.

**Figure 5:**
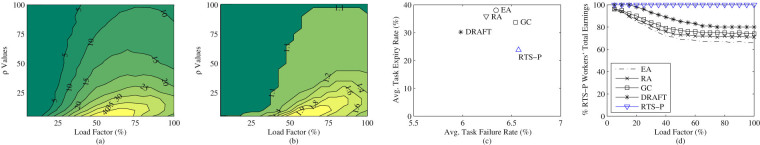
Experimental results under the worker behaviour characteristic setting ${R}_{\mu }^{h}=-$: (**a**) The percentage of all tasks successfully sub-delegated by RTS-P agents; (**b**) The average sub-delegation chain length; (**c**) The average task expiry rates vs. the average task failure rates; (**d**) The total income as a percentage of the total income of RTS-P agents.

As shown in Figs 3(a), 4(a) and 5(a), as LF increases, RTS-P agents sub-delegate an increasing percentage of their workload to other trusted worker agents in the network to mitigate delays for all worker behaviour characteristic settings. If the worker agents are more eager to work as indicated by larger *ρ* values (i.e., worker agents prefer working on their tasks instead of sub-delegating them), fewer tasks are sub-delegated. The highest percentage of tasks is sub-delegated under low general eagerness to work and high workload conditions. As *ρ* increases, this peak sub-delegation percentage shifts towards higher workload conditions. Under ${R}_{\mu }^{h}=+$ (Fig. 3(a)), as LF approaches 100%, an increasing percentage of tasks are sub-delegated. However, when *ρ* values are small and LF values are large (i.e. the general eagerness to work is low while the overall workload is high), the trend reverses. Under ${R}_{\mu }^{h}=0$ (Fig. 4(a)), fewer trustworthy RTS-P agents are able to accommodate new tasks. Thus, there appears to be a systemic “downward shift” of the contour lines in the figure, indicating fewer tasks are being successfully sub-delegated even when agents show high willingness to work (i.e., larger *ρ* values). Under ${R}_{\mu }^{h}=-$ (Fig. 5(a)), workers who produce good results have lower productivity. The systemic downward shift of the contour lines is more pronounced compared to Fig. 4(a). The same trends can be observed for the average sub-delegation chain lengths in Figs 3(b), 4(b) and 5(b). This indicates that as the ${R}_{\mu }^{h}$ value decreases (i.e., the dichotomy between workers’ productivity and quality of work increases), RTS-P adapts its strategy by reducing task sub-delegation throughout the HCN, especially in cases in which workers are highly eager to work and overall workload is high.

The trade-off between the average task failure rates and the average task expiry rates achieved by all five approaches under different ${R}_{\mu }^{h}$ settings is shown in Figs 3(c), 4(c) and 5(c). The overall effect of the RTS-P strategy is to significantly reduce the average task expiry rate (i.e., improving the timeliness of obtaining task results). This comes at the expense of a slightly lower average task result quality. The average task failure rates of RTS-P under all ${R}_{\mu }^{h}$ settings are comparable to those of GC and consistently stay below 7%. The average task expiry rates of RTS-P under all ${R}_{\mu }^{h}$ settings are significantly lower than all other approaches (more than 20% lower than the best performing approach - DRAFT - under the most challenging situation of ${R}_{\mu }^{h}=-$). By making workers sacrifice task quality to a small extent, RTS-P significantly increases the total number of tasks completed by an HCN, putting the *Parrondo’s Paradox* ^31^ to work on a large scale.

Figures 3(d), 4(d) and 5(d) illustrate the total earnings derived by worker agents following the five different approaches under different ${R}_{\mu }^{h}$ settings. The results are averaged over all *ρ* value settings in the experiments. As RTS-P worker agents consistently achieve the highest total earnings, RTS-P is used as the benchmark for all other approaches. It can be observed that the total earnings achieved by EA, RA and GC worker agents as a percentage of those achieved by RTS-P worker agents start to drop under low LF conditions. As LF approaches 100%, EA, RA and GC worker agents achieve around 70% of RTS-P worker agents’ total earnings. DRAFT worker agents earned the same amounts as RTS-P under low LF conditions. The performance of DRAFT worker agents starts to deteriorate under medium LF conditions. As LF approaches 100%, DRAFT worker agents achieve around 75–80% of RTS-P worker agents’ total earnings. Under the challenging situation of ${R}_{\mu }^{h}=-$ (Fig. 5(d)), the advantage of RTS-P over other approaches stalls under high LF conditions. However, when averaged over all LF conditions, RTS-P consistently maintains at least a 14.9% advantage on average over the best performing approach - DRAFT - under all ${R}_{\mu }^{h}$ settings. Overall, RTS-P significantly outperforms existing approaches and its performance is robust in the face of different worker behaviour characteristics.

## Discussion

To summarize, the proposed RTS-P approach leverages Lyapunov stochastic network queueing theory to make joint decisions on task acceptance, sub-delegation, and effort pricing. To our knowledge, RTS-P is the first principled computational approach to assist hierarchical crowdsourcing workers to dynamically sub-delegate tasks and adjust the price of their services based on changing situational factors while ensuring efficient utilization of their collective productivity. High resolution numerical experiments show that RTS-P is robust under various worker behaviour characteristics and significantly outperforms state-of-the-art approaches, especially under conditions of high workload. As recent empirical results show that such conditions are common among crowdsourcing projects^32^, RTS-P can be a useful tool to help HCNs mitigate the adverse effects of herding through efficiently harnessing the available human resources.

Furthermore, a worker can adjust the *ρ*_*i*_(*t*) variable value of his RTS-P agent to take on different roles in a crowdsourcing network. Since each worker can establish his trust relationships with a set of known workers, a worker can focus on tracking workers’ historical performance and building up his list of trusted workers. With such a list, he could reduce the *ρ*_*i*_(*t*) value of his RTS-P agent so as to sub-delegate most of the accepted tasks to other trusted workers, thereby deriving most of his earnings from sub-delegation. By doing so, these workers can serve as task brokerage agents and provide a useful service to the crowdsourcing network. Other workers who are able to spend more time and effort completing tasks can increase the *ρ*_*i*_(*t*) values of their RTS-P agents so as to accept more tasks and sub-delegate only when absolutely necessary, thereby deriving most of their earnings through completing tasks.

RTS-P helps each worker compute a suitable effort price under different situations so that their collective benefits can be maximized. As a task propagates through a sub-delegation chain, subsequent price proposals are subject to Constraint (11) which dictates that workers with prices exceeding the current price for the task being considered for sub-delegation should not be selected (as this will cause the sub-delegator to incur a loss). Thus, there will never be a situation in which a crowdsourcer is forced to accept prices higher than what he can afford. Rather, prices reflect the current demand placed on the workers, and crowdsourcers can decide to either wait or increase their budgets. Such a signal helps coordinate the crowdsourcers’ actions to reduce herding in the crowdsourcing network.

Following this work, we foresee a series of interesting research directions. RTS-P works well for workers who have accumulated some historical performance data in the system. For workers new to a system, there is a large body of literature on reputation bootstrapping^33–35^. Methods from these works can be put in front of RTS-P as a module to build up a system workflow to help new workers build up their track records. The most important direction of this field lies in understanding the dynamics of how the volume of task requests for a worker varies with his reputation and effort pricing. Large-scale user studies in crowdsourcing networks will be needed to investigate this topic. Furthermore, this field will also benefit from more detailed empirical evidence on how workers decide on what types of tasks to accept and what incentive mechanisms are effective.

In conclusion, the proposed approach and results provide a stepping stone towards more efficient management of large-scale hierarchical crowdsourcing based on evidence about workers’ behaviours, and ultimately help improve the collective productivity of our connected world.
